# Supplementary material for: Anti-Melanogenic Effects of Cnidium monnieri Extract via p38 Signaling-Mediated Proteasomal Degradation of Tyrosinase
Source: Plants (Basel). 2024 May 9;13(10):1305. doi: 10.3390/plants13101305 (PMC11125256; doi:10.3390/plants13101305)
Supplement: Supplementary file 1 [file plants-13-01305-s001.zip › plants-2983743-supplementary.pdf]

Supplementary Materials **Plants**

Anti-melanogenic effects of *Cnidium monnieri* extract via p38 signaling-mediated proteasomal degradation of tyrosinase

Soon Ho Choi[1,†], Hyunggun Kim[2,†], Jeon Hwang-Bo[3], Kyoung Mi Kim[4], Jeong Eun Kwon[3], Sung Ryul Lee[5], Sun Ha Hwang[3], Se Chan Kang[3,*] and Yeong-Geun Lee[3,*]

[1] Research Institute, APRG Inc., Yongin 16950. Republic of Korea.

[2] Department of Biomechatronic Engineering, Sungkyunkwan University, Suwon 16419, Republic of Korea

[3] Department of Biopharmaceutical Biotechnology and Graduate School of Biotechnology, Kyung Hee University, Yongin 17104, Republic of Korea

[4] Research Center, CureBio Therapeutics Co., Ltd., Suwon 16229, Republic of Korea

[5] Department of Convergence Biomedical Science, Cardiovascular and Metabolic Disease Center, College of Medicine, Inje University, Busan, 47392, Republic of Korea

*Correspondence: sckang@khu.ac.kr (S.C.K.); lyg629@nate.com (Y.-G.L.)

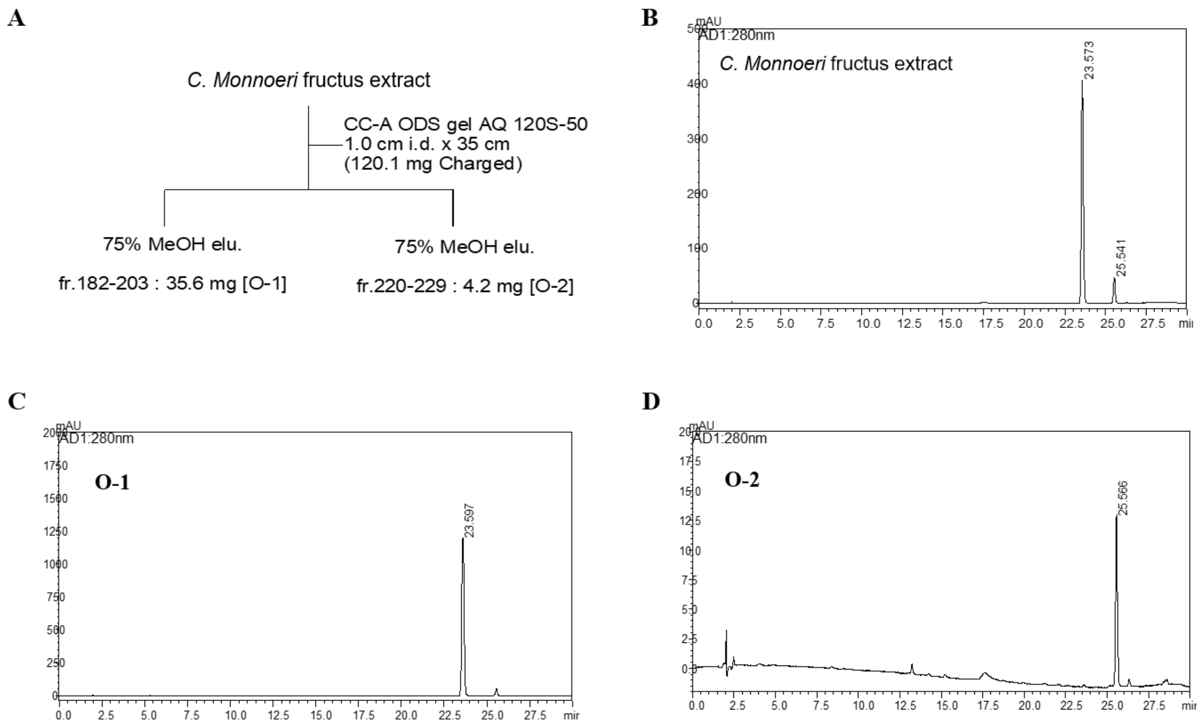

Supporting information Figure s1. Isolation procedure of *C. Monnoeri* fructus extract and compound of *C. Monnoeri* fructus extract fractionated by semi-preparative HPLC. (A) Isolation procedure of *C. Monnoeri* fructus extract. (B-D) Compounds of *C. Monnoeri* fructus extract fractionated by semi-preparative HPLC. The flow rate of the mobile phase was maintained at 5, and the compound was detected at 280 nm. The main substance was separated at Rt 23.6 min and named O-1.

Supporting information table s1. NMR data of O-1 in $CDCl_3$ (600 MHz)

No.	O-1 ($CDCl_3$, 600 MHz)		Osthol ($CDCl_3$, 400 MHz)	
	δ_H (J in Hz)	δ_C, mult.	δ_H (J in Hz) [1]	δ_C, mult. [2]
2	-	161.4	-	161.3
3	6.25 (d, 9.0)	112.8	6.25 (d, 9.4)	112.9
4	7.63 (d, 9.0)	143.7	7.61 (d, 9.4)	143.8
4a	-	113.0	-	113.0
5	7.31 (d, 8.4)	126.2	7.29 (d, 8.7)	126.2
6	6.85 (d, 8.4)	107.3	6.83 (d, 8.7)	107.4
7	-	160.2	-	160.2
8	-	118.0	-	117.9
8a	-	152.8	-	152.8
1'	3.55 (d, 6.6)	21.9	3.52 (d, 7.4)	21.9
2'	5.25 (d, 6.6)	121.1	5.22 (t, 7.4)	121.2
3'	-	132.7	-	132.6
4'	1.86 (s)	25.8	1.84 (s)	25.8
5'	1.69 (s)	17.9	1.67 (s)	17.9
7-OCH_3	3.94 (s)	56.1	3.92 (s)	56.0

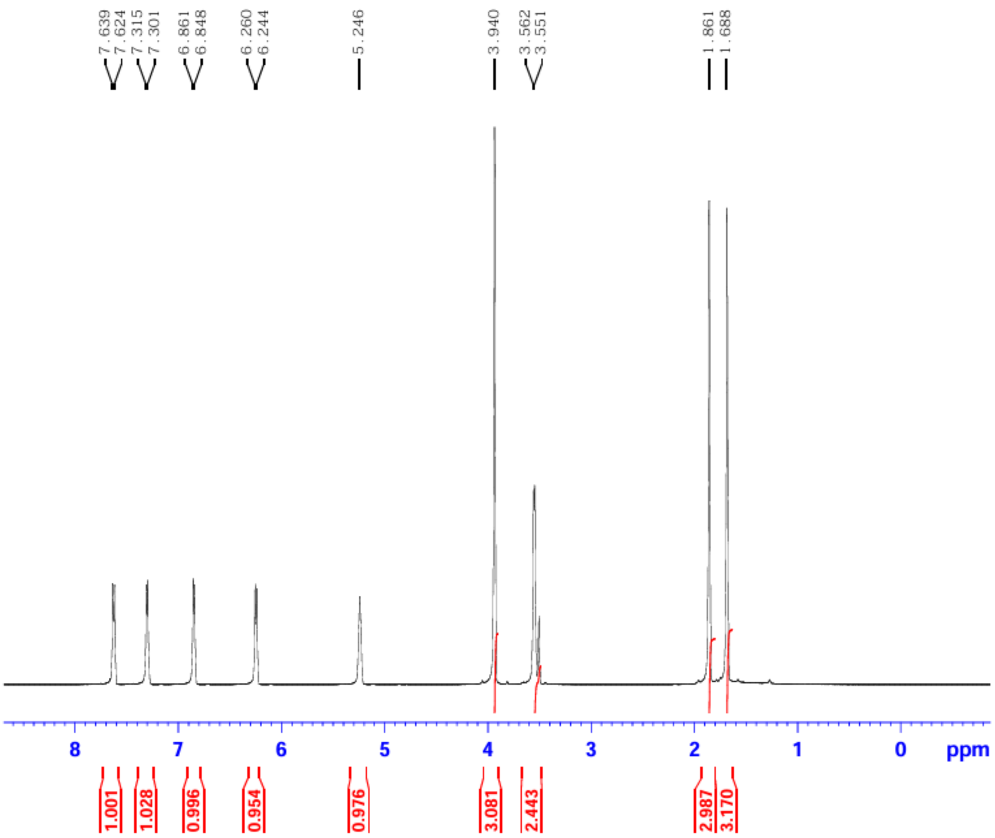

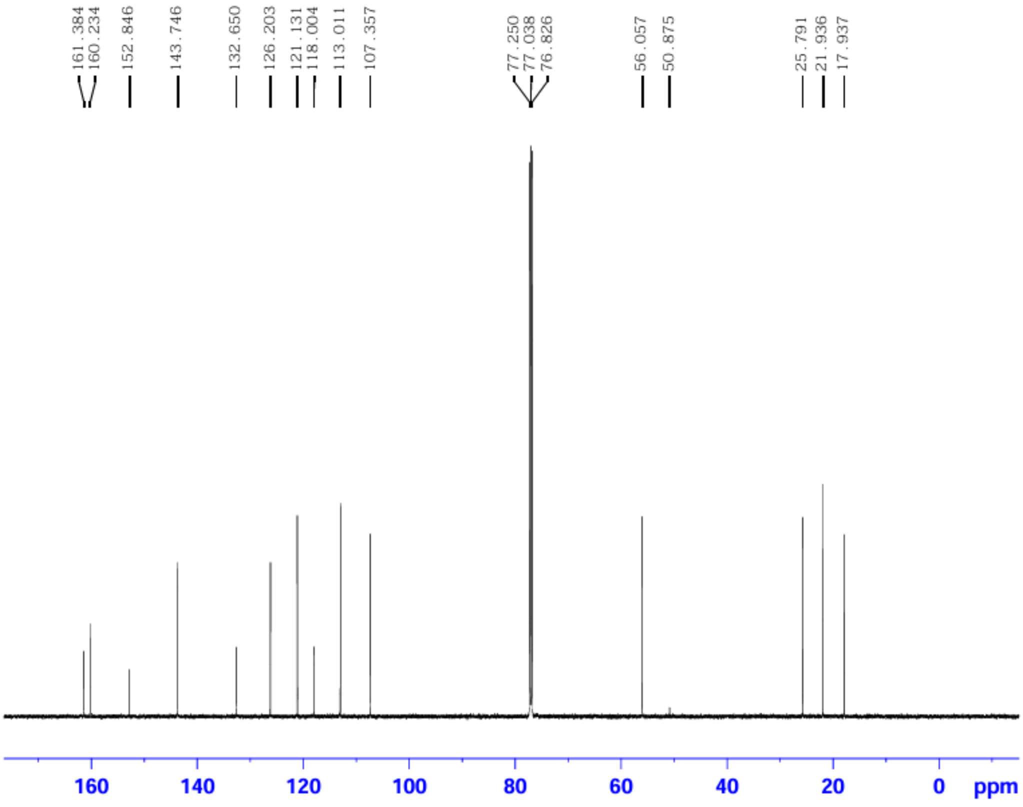

Supporting information Figure s2. 1D NMR data for osthol (600 MHz, $CDCl_3$)

Reference

1. ITO, C.; FURUKAWA, H. Constituents of *Murraya exotica* L. Structure elucidation of new coumarins. *Chem. Pharm. Bull.* **1987**, *35(10)*, 4277-4285.

2. Zhou, P.; Takaishi, Y.; Duan, H.; Chen, B.; Honda, G.; Itoh, M.; Takeda, Y.; Kodzhimatov, O.K.; Lee, K.H. Coumarins and bicoumarin from *Ferula sumbul*: anti-HIV activity and inhibition of cytokine release. *Phytochemistry* **2000**, *53(6)*, 689-697.
